# Nutritional Asymmetries Are Related to Division of Labor in a Queenless Ant

**DOI:** 10.1371/journal.pone.0024011

**Published:** 2011-08-23

**Authors:** Chris R. Smith, Andrew V. Suarez, Neil D. Tsutsui, Sarah E. Wittman, Benjamin Edmonds, Alex Freauff, Chadwick V. Tillberg

**Affiliations:** 1 Department of Biology, Earlham College, Richmond, Indiana, United States of America; 2 Departments of Animal Biology and Entomology, University of Illinois, Urbana, Illinois, United States of America; 3 Department of Environmental Science, Policy and Management, University of California, Berkeley, California, United States of America; 4 Department of Botany, La Trobe University, Melbourne, Victoria, Australia; 5 Department of Biology, Linfield College, McMinnville, Oregon, United States of America; Royal Holloway University of London, United Kingdom

## Abstract

Eusocial species exhibit pronounced division of labor, most notably between reproductive and non-reproductive castes, but also within non-reproductive castes via morphological specialization and temporal polyethism. For species with distinct worker and queen castes, age-related differences in behavior among workers (e.g. within-nest tasks versus foraging) appear to result from physiological changes such as decreased lipid content. However, we know little about how labor is divided among individuals in species that lack a distinct queen caste. In this study, we investigated how fat storage varied among individuals in a species of ant (*Dinoponera australis*) that lacks a distinct queen caste and in which all individuals are morphologically similar and capable of reproduction (totipotent at birth). We distinguish between two hypotheses, 1) all individuals are physiologically similar, consistent with the possibility that any non-reproductive may eventually become reproductive, and 2) non-reproductive individuals vary in stored fat, similar to highly eusocial species, where depletion is associated with foraging and non-reproductives have lower lipid stores than reproducing individuals. Our data support the latter hypothesis. Location in the nest, the probability of foraging, and foraging effort, were all associated with decreased fat storage.

## Introduction

Division of labor is a hallmark of eusocial behavior. In the eusocial Hymenoptera, the primary division of labor is between the reproductive individuals (the queens) and the female work force. Among the workers, further task subdivisions may also exist based either on morphological specialization or age related changes in behavior (temporal polyethism). This division of labor and resulting task allocation can increase colony efficiency, which ultimately translates into reproduction and the foundation of daughter colonies [1].

Variation in nutrition among individuals is a conserved mechanism regulating castes and division of labor in social insects, both between reproducers and non-reproducers and among non-reproducers. While many factors are known to differ between and within castes such as lifespan [2]–[6], genetics [7]–[12], and encounter rate [13], among others, variation in fat content is consistently different between queens and workers [14] and is associated with the transition from nest work to foraging in many social insects workers [15]. Queens are more corpulent than workers and nest workers are more corpulent than foragers. The depletion of stored fat is gradual in workers and typically correlates with a movement away from the brood (nursing) and eventually out of the nest (foraging). Toth et al. [16] demonstrated a causal link between fat storage and the onset of foraging behavior in honeybees by applying an inhibitor of lipid storage to bees, which increased the likelihood of precocious foraging (though social context was still important).

Most studies documenting a link between nutrition or physiological state and division of labor are on highly eusocial species with morphologically distinct castes. It is unclear how nutritional status may affect either reproductive and/or foraging divisions of labor in a species without morphologically distinct castes and where all individuals are at least capable of reproducing (totipotent at birth). Some species of ant, especially in the poneroid clade [17]–[18], have no physical differentiation between queens and workers and reproductive division of labor is maintained by behavioral dominance hierarchies. That is, all females in the nest are, at the onset of adulthood, fully capable of mating and reproducing. Data on *Dinoponera*, the queenless ant we studied, and other queenless ants suggest that dominance hierarchies are relatively short, with only one (in the case of *Dinoponera*) or few reproducers [17]–[19]. Further, the reproducer(s) can be chemically distinct from nest mates and likely mark their eggs with their scent [20]–[21], enabling the reproducer or nest mates to destroy eggs laid by others (and the “pretending” reproducer can be violently punished)[22].

We questioned whether societies of the queenless ant, *Dinoponera australis*, still have a nutritionally based division of labor or whether individuals maintain their physical condition (and fat reserves) in order to more effectively compete for reproductive opportunities (i.e., maintaining their capacity as a “hopeful reproductive”). Nutritional inequalities may reinforce or be reinforced by dominance interactions, thus stabilizing reproductive division of labor and maintaining the link between physiological state (fat content) and foraging behavior. We evaluated two alternative hypotheses: 1) all individuals are physiologically similar, consistent with the possibility that any worker may eventually become reproductive, and 2) depletion of fat stores is associated with movement away from brood and an eventual transition to foraging.

## Methods

We conducted all field-work and sample collections at Iguazú National Park, Misiones Province, in northeastern Argentina. The habitat consists of subtropical rainforest, receiving approximately 1800 mm of rain per year. Our field sites were located in the forest near the CIEN (Centro de Investigaciones Ecológicas Subtropicales) station. The work was conducted in December, 2005, and January, 2009.

*Dinoponera australis* colonies were observed for several hours each and all foragers were marked with enamel paint. In one colony, paint markings were unique for each individual and the total number of foraging trips was counted per individual (over two days). We excavated five entire nests (one in December 2005, four in January 2009). Prior to excavation, we collected foragers near the entrance of the nests as they were leaving or returning to the nest. During excavation, we collected all individuals, including adults, pupae, brood, and eggs, from each nest chamber into separate containers and measured the depth of the chamber from the soil surface. Colonies were excavated by first digging a pit next to the colony entrance and then the shafts and chambers of the nest were exposed by gradually digging from the top in cross-section. While it is possible that individuals naturally occurring at different depths mix during excavation, studies on harvester ants [23], at least, suggest that this methodology does not result in a great deal of vertical mixing of individuals. To account for differences in total depth among different nests, we adjusted this value by calculating relative depth (chamber depth divided by total nest depth). To ensure that we had discovered all of the chambers, we continued to dig at least 20 cm beyond the last chamber, following any possible tunnels to their ends, down to 1.5 m in depth.

All samples were sacrificed by freezing, followed immediately by desiccation in a low-temperature oven (50°C). We measured total dry mass of all adult samples and then performed fat extraction using a Soxlet extractor and ether as the solvent [24], followed by another measurement of the mass for each individual (lean mass). The proportion of fat mass for each individual was calculated as the individual's fat mass (dry mass – lean mass) divided by its initial dry mass. Finally, to determine size differences among individuals in the colony, we measured head width at the widest point.

We used ANCOVA to test whether relative position in the nest predicted fat content or head size, whether colonies differed in each variable, and whether the slopes differed between colonies. Fat content data were collected for 5 colonies whereas head width for only the four colonies collected in 2009. Logistic regression was used to examine how the probability of foraging changed with increased fat content and body size. Finally, we used a regression to examine whether fat content predicted foraging effort (the number of observed foraging trips) for one colony.

## Results

**Figure 1:**
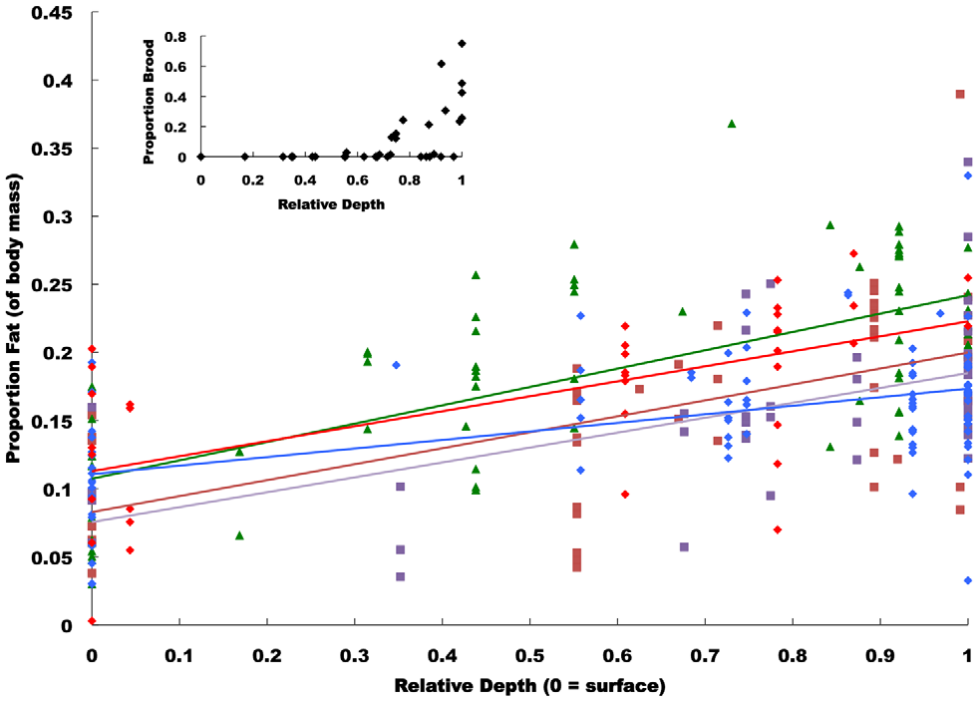
The relationship between individual fat content (as a proportion of total dry body mass) and the relative depth at which the individual was collected (1 being deepest and 0 being the surface) for five colonies of *Dinoponera australis* (each colony is coded by a different color). Colonies differed in the slope of the relationship between fat content and relative depth (F_4,267_ = 3.41, P = 0.01), but the relationship is highly significant (F_1,267_ = 137.93, P<0.0001) and colonies did not differ in average individual fat content (F_4,267_ = 1.27, P = 0.28). The inset shows brood (larvae and pupae) as a function of depth; only the deepest chambers contained brood.

Of the five colonies collected in this study, the average number of workers per colony was 57, with a range of 36–86 (Table 1). On average, we sampled 41 individuals that had not been observed foraging and 15 foragers per nest (Table 1). *D. australis* colonies did differ in the slope of the relationship between fat content and depth (F_4,267_ = 3.41, P = 0.01, Fig. 1), but not in head width and depth (F_3,231_ = 1.53, P = 0.21); thus, we used a separate slopes model to examine differences in fat content across colonies. For all colonies, individual corpulence increased with depth (F_1,267_ = 137.93, P<0.0001), but head size was not associated with depth (F_1,231_ = 2.76, P = 0.10). Colonies did not differ in average fat content (F_4,267_ = 1.27, P = 0.28), but did differ in average head width (F_3,231_ = 4.99, P = 0.002). The most corpulent individuals in each nest were found in chambers with brood and were in or near the deepest chamber (Fig. 1).

**Table 1:** Descriptive statistics for each colony.

Colony #	Colony Size	Prop. Fat (N)	Head Width (mm)	# Foragers	# Pupae	# Larvae
5	36	0.17 +/− 0.01 (36)	---	15	---	---
6	46	0.15 +/− 0.01 (41)	5.09 +/− 0.02 (41)	13	52	8
15	48	0.16 +/− 0.01 (48)	5.19 +/− 0.02 (48)	6	33	0
17	70	0.19 +/− 0.01 (70)	5.15 +/− 0.02 (70)	19	66	12
23	86	0.15 +/− 0.01 (83)	5.24 +/− 0.01 (81)	21	71	1

Fat content is as a proportion of total dry body mass. Fat content and head width are reported as means +/- standard error with sample size in parentheses. Brood was present but not counted for colony 5. Colony 5 was collected in 2004 while the remainder in 2009.

**Figure 2:**
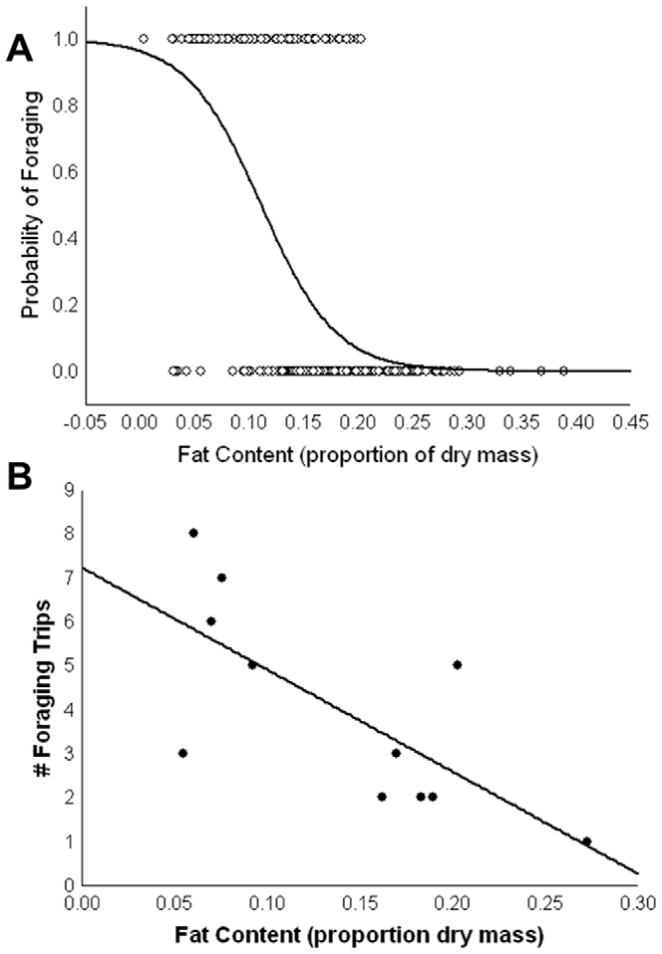
The probability of foraging (panel a; χ^2^ = 102.86, P<0.0001) and the amount of foraging effort (panel b; F_1,9_ = 9.71, P = 0.012) are significantly predicted by fat content (as a proportion of total dry body mass) in *Dinoponera australis*.

The probability of foraging was significantly predicted by fat content (χ^2^ = 102.86, P<0.0001, Fig. 2a), but not head width (χ^2^ = 1.66, P = 0.2). No foraging was observed by individuals with more than 25% of their total dry mass stored as fat, and the most corpulent individuals in four of five colonies had >33% fat, 27% in the fifth colony (Fig. 1). The probability of foraging was 50% when fat content declined to ∼12% and reached ∼90% when fat content was 5% or less. Furthermore, in agreement with the above, fat content was a significant predictor of the number of foraging trips, such that decreasing fat content predicted increasing foraging effort (F_1,9_ = 9.71, P = 0.012, R^2^ = 0.52, Fig. 2b).

## Discussion

Our results support the hypothesis that division of labor can be organized by nutritional status, and that fat storage may be a conserved means of organizing foraging behavior even in a species where all individuals are capable of mating and reproducing. Even when all individuals are capable of reproducing, nutritional variation may sort individuals into distinct groups and physiological cues conserved from solitary insects (foraging when fat storage is low) may reinforce specialization. Studies of the molecular basis of foraging and reproduction in social insects generally suggest that division of labor is derived from conserved pathways present in solitary ancestors [25]–[26].

We observed a wide range of fat content (as a percentage of total dry body mass), from less than 1% to 39% (137 fold increase from least to most corpulent). Fat content varied with both the relative depth at which individuals were found in the nest (Fig. 1) and the probability of foraging and number of foraging trips made by individuals (Fig. 2). These results are in agreement with data from honeybees where fat storage has a causal role in regulating the timing of foraging behavior [16]. Starvation has also been shown to increase foraging in some primitively eusocial wasps [27]. Future studies that manipulate individual nutritional status in *D. australis*, however, are necessary to determine the causality of this relationship; clearly, decreased fat storage may be a consequence rather than cause of foraging behavior. As in solitary insects, *D. australis* workers increase foraging when they have depleted fat stores [28]. Similar to ants where workers have no hope of reproduction, *D. australis* foragers do not maintain their body condition, even though they theoretically can mate and reproduce. It is also selectively advantageous, for colonies, when the most energetically depleted individuals undertake the most dangerous tasks, such as foraging, because the loss of these individuals represents a less severe energetic loss to the colony [4].

Head width is a standard proxy of body size in ants [29] and size can influence competition for reproductive opportunities [30]. Assuming that increased fat content is predictive of greater reproductive potential, we found no evidence that head width plays a large role in access to reproduction as has been found in some ants and other social insects [31–32, but not 33], though the most corpulent individuals in each nest (putative reproducers – gamergates) were above average in head width for their colony. Other studies (across social insects) have found an influence of age on dominance and access to reproduction in societies without morphologically distinct queens [34]–[36]; younger individuals tend to be at the top of the dominance hierarchy. In many social insects, age, fat content, and ovarian status all co-vary and are related to the transition from in-nest behaviors to foraging [4], with older, leaner, individuals with degenerated ovaries being more likely to forage. Therefore, our data are consistent with data from *Dinoponera quadriceps* [34] showing that young individuals are at the top of the dominance hierarchy. Similarly, our data are consistent with those from another queenless ant, *Streblognathus peetersi*, in which more dominant individuals have greater vitellogenin titres in their haemolymph [37]; vitellogenin is an yolk egg precursor that is stored in the fat bodies.

In *Dinoponera quadriceps*, dominance hierarchies tend to be relatively short where only few individuals actively compete for reproduction [34]. Monnin and Ratnieks [19] developed a model that predicts short dominance hierarchies in *D. quadriceps* because increased hierarchy length is likely to decrease overall colony productivity (higher levels of antagonism over reproduction results in much less work being done in the colony). Our data generally support the predictions of this model in that most workers are likely to be too deficient in fat stores to reproduce. If individuals not predicted to be foragers (probability of foraging is zero in the logistic regression, Fig. 2a) are considered to be participating in the dominance hierarchy, then our data suggest a hierarchy length of 4.6 individuals (9% of the colony), double that estimated by Monnin and Ratnieks [19], though their estimate of *D. australis* colony size (13 workers [38]) is four times lower than our estimate (53 workers). A hierarchy length of 4.6 is, however, in line with their estimate for *D. quadriceps*, which has a colony size of 89 workers.

These results suggest that nutritional status is correlated with division of labor in an ant society lacking distinct queen and worker castes. Less corpulent individuals are those most likely to forage while those with the greatest fat reserves are nearer the brood and potential reproductive opportunities. These results highlight the conservation of nutrition as a potential organizer of division of labor across multiple origins of sociality, from queenless societies, such as *Dinoponera*, to the complex societies of honey bees [15] and fire ants [3].

## References

[1] Oster GF, Wilson EO (1978). Caste and ecology in the social insects..

[2] Tschinkel WR (1987). Seasonal life history and nest architecture of a winter-active ant, *Prenolepis imparis*.. Insect Soc.

[3] Tschinkel WR (1993). Sociometry and sociogenesis of colonies of the fire ant *Solenopsis invicta* during one annual cycle.. Ecol Monogr.

[4] Hölldobler B, Wilson EO (1990). The Ants..

[5] Morón D, Witek M, Woyciechowski M (2008). Division of labour among workers with different life expectancy in the ant *Myrmica scabrinodis*.. Anim Behav.

[6] Münch D, Amdam GV, Wolschin F (2008). Aging in a eusocial insect: molecular and physiological characteristics of life span plasticity in the honey bee.. Functional Ecology.

[7] Stuart RJ, Page RE (1991). Genetic component to division of labor among workers of a Leptothoracine ant.. Naturwiss.

[8] Julian GE, Fewell JH (2004). Genetic variation and task specialization in the desert leaf-cutter ant, *Acromyrmex versicolor*.. Anim Behav.

[9] Schwander T, Rosset H, Chapuisat M (2005). Division of labor and worker size polymorphism in ant colonies: the impact of social and genetic factors.. Behav Ecol Sociobiol.

[10] Tilley CA, Oldroyd BP (1997). Unequal subfamily proportions among honey bee queen and worker brood.. Anim Behav.

[11] Kerr WE (1950). Genetic determinants of castes in the genus *Melipona*.. Genetics.

[12] Hayashi Y, Lo N, Miyata H, Kitade O (2007). Sex-linked genetic influence on caste determination in a termite.. Science.

[13] Gordon DM, Mehdiabadi NJ (1999). Encounter rate and task allocation in harvester ants.. Behav Ecol Sociobiol.

[14] Smith CR, Tschinkel WR (2006). The sociometry and sociogenesis of reproduction in the Florida harvester ant, *Pogonomyrmex badius*.. J Insect Sci.

[15] Toth AL, Robinson GE (2005). Worker nutrition and division of labour in honeybees.. Anim Behav.

[16] Toth AL, Kantarovich S, Meisel AF, Robinson GE (2005). Nutritional status influences socially regulated foraging ontogeny in honey bees.. J Exp Biol.

[17] Peeters C, Choe J, Crespi B (1997). Morphologically ‘primitive’ ants: comparative review of social characters, and the importance of queen-worker dimorphism.. The Evolution of Social Behaviour in Insects and Arachnids.

[18] Monnin T, Peeters C (2008). How many gamergates is an ant queen worth?. Naturwiss.

[19] Monnin T, Ratnieks FLW (1999). Reproduction versus work in queenless ants: when to join a hierarchy of hopeful reproductives?. Behav Ecol Sociobiol.

[20] Peeters C, Monnin T, Malosse C (1999). Cuticular hydrocarbons correlated with reproductive status in a queenless ant.. Proc R Soc Lond B.

[21] Monnin T, Malosse C, Peeters C (1998). Solid Phase MicroExtraction and cuticular hydrocarbon differences related to reproductive activity in the queenless ant *Dinoponera quadriceps*.. J Chem Ecol.

[22] Monnin T, Ratnieks FLW, Jones G, Beard R (2002). Pretender punishment induced by chemical signalling in a queenless ant.. Nature.

[23] Tschinkel WR (1998). Sociometry and sociogenesis of colonies of the harvester ant, *Pogonomyrmex badius*: worker characteristics in relation to colony size and season.. Insectes Soc.

[24] Smith CR, Tschinkel WR (2009). Ant fat extraction with a Soxhlet extractor.. Cold Spring Harb Protoc.

[25] Smith CR, Toth AL, Suarez AV, Robinson GE (2008). Genetic and genomic analyses of the division of labour in insect societies.. Nat Rev Genet.

[26] Toth AL, Robinson GE (2007). Evo-devo and the evolution of social behavior.. Trends Genet.

[27] Markiewicz DA, O'Donnell S (2001). Social dominance, task performance and nutrition: implications for reproduction in eusocial wasps.. J Comp Physiol A.

[28] Simpson SJ, Raubenheimer D (1993). The central role of the haemolymph in the regulation of nutrient intake in insects.. Physiol Entomol.

[29] Kaspari M, Weiser MD (1999). The size-grain hypothesis and interspecific scaling in ants.. Funct Ecol.

[30] Sullivan JD, Strassman JE (1984). Physical variability among nest foundresses in the polygynous social wasp, *Polistes annularis*.. Behav Ecol Sociobiol.

[31] Tibbetts EA, Shorter JR (2009). How do fighting ability and nest value influence usurpation contests in *Polistes* wasps?. Behav Ecol Sociobiol.

[32] Heinze J, Oberstadt B (1999). Worker age, size and social status in queenless colonies of the ant *Leptothorax gredleri*.. Anim Behav.

[33] Bourke AFG (1988). Dominance orders, worker reproduction, and queen-worker conflict in the slave-making ant *Harpagoxenus sublaevis*.. Behav Ecol Sociobiol.

[34] Monnin T, Peeters C (1999). Dominance hierarchy and reproductive conflicts among subordinates in a monogynous queenless ant.. Behav Ecol.

[35] Cuvillier-Hot V, Gadagkar R, Peeters C, Cobb M (2002). Regulation of reproduction in a queenless ant: aggression, pheromones and reduction in conflict.. Proc R Soc Lond B.

[36] Hughes CR, Strassman JE (1988). Age is more important than size in determining dominance among workers in the primitively eusocial wasp, *Polistes instabilis*.. Behaviour.

[37] Cuvillier-Hot V, Lenoir A, Crewe R, Malosse C, Peeters C (2004). Fertility signaling and reproductive skew in queenless ants.. Anim Behav.

[38] Paiva RVS, Brandão CRF (1995). Nests, population, and reproductive status of workers in the true giant queenless ponerine ant *Dinoponera* Roger (Hymenoptera: Formicidae).. Ethol Ecol Evol.

